# Spatially-Resolved Proteomics: Rapid Quantitative Analysis of Laser Capture Microdissected Alveolar Tissue Samples

**DOI:** 10.1038/srep39223

**Published:** 2016-12-22

**Authors:** Geremy Clair, Paul D. Piehowski, Teodora Nicola, Joseph A. Kitzmiller, Eric L. Huang, Erika M. Zink, Ryan L. Sontag, Daniel J. Orton, Ronald J. Moore, James P. Carson, Richard D. Smith, Jeffrey A. Whitsett, Richard A. Corley, Namasivayam Ambalavanan, Charles Ansong

**Affiliations:** 1 Biological Science Division, Pacific Northwest National Laboratory, Richland, WA 99352, USA; 2 Department of Pediatrics, University of Alabama at Birmingham, Birmingham, AL 35249, USA; 3 Division of Pulmonary Biology, Cincinnati Children’s Hospital Medical Center, Cincinnati, OH 45229, USA; 4 Texas Advanced Computing Center, University of Texas at Austin, Austin, TX 78712, USA

## Abstract

Laser capture microdissection (LCM)-enabled region-specific tissue analyses are critical to better understand complex multicellular processes. However, current proteomics workflows entail several manual sample preparation steps and are challenged by the microscopic mass-limited samples generated by LCM, impacting measurement robustness, quantification and throughput. Here, we coupled LCM with a proteomics workflow that provides fully automated analysis of proteomes from microdissected tissues. Benchmarking against the current state-of-the-art in ultrasensitive global proteomics (FASP workflow), our approach demonstrated significant improvements in quantification (~2-fold lower variance) and throughput (>5 times faster). Using our approach we for the first time characterized, to a depth of >3,400 proteins, the ontogeny of protein changes during normal lung development in microdissected alveolar tissue containing only 4,000 cells. Our analysis revealed seven defined modules of coordinated transcription factor-signaling molecule expression patterns, suggesting a complex network of temporal regulatory control directs normal lung development with epigenetic regulation fine-tuning pre-natal developmental processes.

Proteomics studies are typically performed on whole tissues or organs allowing for the detection and the quantification of thousands of proteins from a single liquid chromatography-mass spectrometry (LC-MS) analysis. While this approach has proven useful in helping to extend current biological knowledge, proteomes generated from lysates of whole tissues or organs represent a blend of cells from disparate anatomical regions with diverse cell subpopulations in different cellular contexts, unavoidably producing an averaging effect that hinders the elucidation of deeper biological insights. Thus, it is now becoming increasingly recognized that to gain a deeper, more refined understanding of complex biological processes such as normal development of multi-cellular organisms and the aberrant states from which diseases arise, global protein profiling of specific spatially defined regions and/or cell types of tissues (i.e., spatially-resolved proteomics) is required and essential^1^,^2^,^3^.

Mass spectrometry imaging approaches are promising and have been used to provide spatial localization information on proteins in tissues, however the extent of proteome coverage is limited to a few hundred identified proteins and prone to matrix effects challenging quantification^4^,^5^. Laser capture microdissection (LCM) is an elegant approach that enables microscopic isolation of specific cell types or defined regions from a tissue preserving crucial spatial information, which is not available when dispersed cells from the tissue are used. However, LCM-enabled spatially-resolved proteomics is challenged by the small, mass-limited samples generated by LCM, typically on the order of a microgram of protein or less. Advancements in commercial mass spectrometry instrumentation^6^,^7^ and informatics^8^,^9^ now allow effective label-free quantitative proteomics. Application towards microscopic LCM samples, however, fails to benefit from these improvements, because sample preparation/handling results in significant samples losses and poor reproducibility. Indeed the vast majority of bottom-up proteomics sample preparation/handling workflows are performed manually, include numerous steps and subsequently are subject to considerable sample losses, contamination, low throughput and variability^10^,^11^,^12^,^13^. For example, the non-specific adsorption of biological material on surfaces is a well-known problem^14^ and becomes more problematic as the protein amount is reduced^15^. Hence, new approaches are critical and necessary for effective spatially-resolved proteomics at microscopic scale. Recent studies have shown that the reduction of sample handling improves the sensitivity and the reproducibility of subsequent proteomics analysis for ultrasensitive global proteome measurements (defined as less than 5,000 cells starting material and proteome coverage more than 3,000 proteins)^10^,^13^,^16^. Wiśniewski *et al*. developed the filter-aided sample preparation (FASP) protocol, now widely utilized. In this approach, all the steps of sample preparation are performed in the same vessel, a filtration unit, which acts as ‘proteomic reactor’^17^. Recently, this ‘proteomic reactor’ strategy has been modified to various supports such as a stop-and-go extraction tip^11^, or by using paramagnetic beads to immobilize the sample inside a single test-tube during all the preparation steps^16^. Nevertheless, these proteomic reactor strategies require several manual steps during sample processing/handling and entail long processing times, challenging throughput, reproducibility and quantification accuracy for ultrasensitive proteomics applications^18^,^19^,^20^.

The potential of immobilized enzyme reactors (IMERs) for providing rapid protein digestion has been broadly established in the literature^21^,^22^. Furthermore, a variety of different configurations employing IMERs have convincingly demonstrated compatibility with low protein loadings under 1 μg^23^,^24^. Here we efficiently couple LCM to a nanoproteomics IMER platform augmented with increased LC operating pressure and reduction of column internal diameter. Thus decreasing sample size requirements and consumption, while increasing ionization efficiency^25^,^26^ for better sensitivity and quantification relative to a prior iteration^27^ enables the LCM work described here. The platform employs online digestion and desalting that reduces manual sample handling to the extreme with only a single manual sample handling step that allows for the analysis of sub-microgram quantities of proteins with high reproducibility and throughput. Benchmarking against the current state of the art in ultrasensitive global proteomic analysis approaches demonstrated significant improvements in quantification accuracy (approximately 2-fold lower coefficient of variation) and throughput (more than 5 times faster); suggesting that our LCM-proteomics platform provides a powerful approach for enabling spatially-resolved ultrasensitive proteomics analyses in clinical and developmental biology applications where quantification accuracy, sensitivity, and throughput are critical.

Proper lung development and function are essential for terrestrial life. In the lung, the critical functions of gas exchange and production of lung surfactant are performed by cells in the alveoli, tiny anatomical structures localized at the termini of the branched airways^28^,^29^. Here we applied our LCM-proteomics platform to profile the alveolar proteome during normal lung development from laser capture micro-dissected alveolar parenchymal samples containing 4,000 cells. While a number of gene expression analyses describing normal lung development have been reported^30^,^31^, there is only a single report of normal lung development at the proteome level utilizing whole tissue lysate^30^,^32^. Using our LCM-proteomics platform the proteome here is profiled to a depth of more than 3,400 proteins across three developmental time-points relevant to alveologenesis including 350 transcription factors and signaling molecules. The results enabled a first protein-level view of coordinated transcription regulator-signaling molecule expression. The demonstrated utility of our high throughput LCM-proteomics measurement strategy for gaining biological insight should promote broader adoption and application of spatially-resolved proteomics approaches^33^.

## Results and Discussion

### A high-throughput LCM-proteomics platform for ultrasensitive analysis

**Figure 1:**
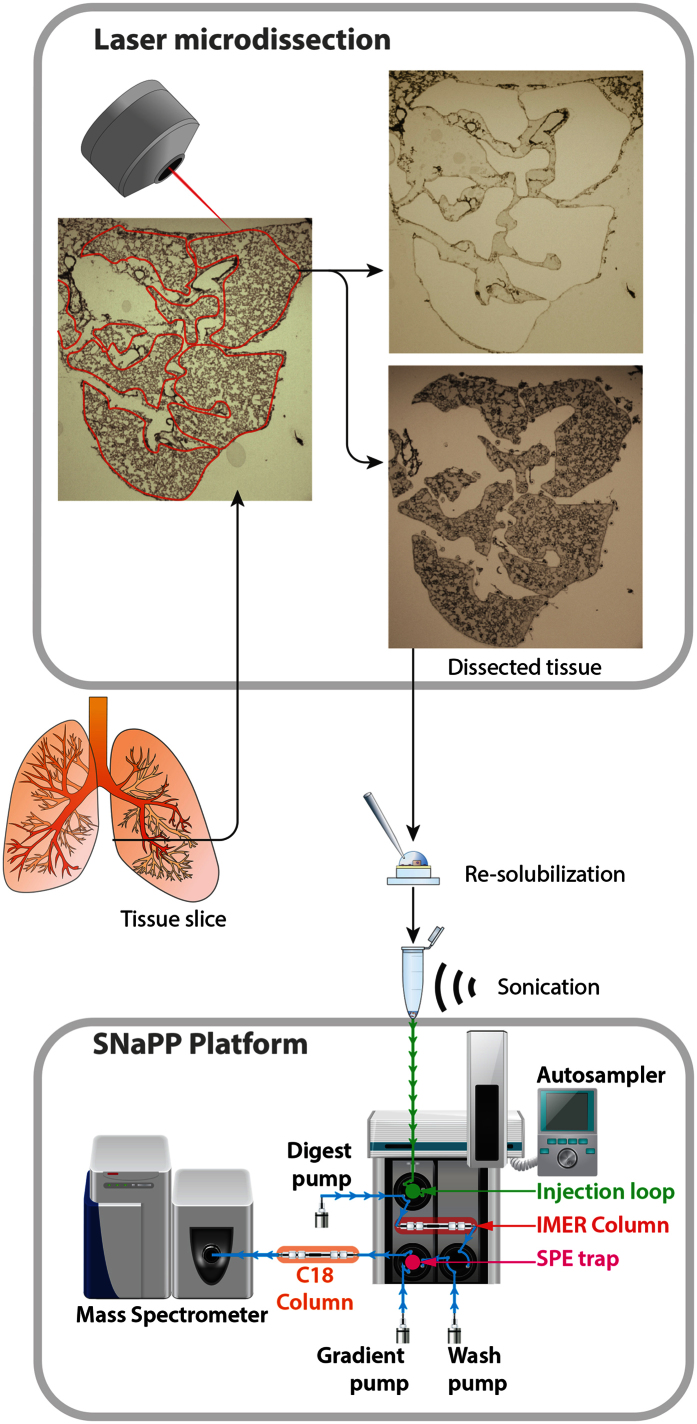
High-throughput LCM-proteomics platform for ultrasensitive analysis. Schematic of the LCM-proteomics workflow. Lung tissue is sliced and the microdissection is performed on a slice. The top panel is a representative LCM cut of the alveolar tissue from a sample obtained at postnatal day 7. The left image is the schematic of the cutout. The top-right image is the tissue leftover after the cutout; the bottom-right image is showing the tissue excised onto the LCM cap. The protein are then extracted from the microdissected tissue and injected onto the IMER column for on-line proteolysis. Contaminating substances are removed via on-line SPE trap and neat peptide is transferred on-line to C18 analytical column for MS analysis. “*LCM-Prot; LCM-proteomics platform*”.

There is considerable interest in proteomic analysis of LCM dissected tissues, as they promise to offer spatially-resolved insights into tissue-specific mechanisms and signaling that are obscured in bulk proteomics^1^,^11^,^33^. However, current bottom-up proteomics workflows require several manual processing steps to generate peptides for LC-MS/MS analysis, incurring significant sample losses and subsequently are not readily amenable to the minute samples generated by LCM. Furthermore, each step in the analytical process has an associated technical variability and their contributions to the total variance are multiplicative^34^. In order to address the above challenges, improve the robustness and the sensitivity of bottom-up proteomics of LCM samples, and enable routine, reproducible, high throughput spatially-resolved proteomics we describe here an approach that efficiently couples LCM to a nanoproteomics IMER platform. The simple platform design we present comprises a commercially available IMER digestion column and solid phase extraction desalting column (SPE) directly connected to a commercial LC system and interfaced with a Q-Exactive mass spectrometer. Importantly the platform is augmented with a doubling of the LC operating pressure and a 2-fold reduction of the analytical column volume relative to an earlier variant we described prevously^27^ improving measurement sensitivity (Fig. 1). The platform employs a minimalist concept for sample handling where following sample resolubilization (in a denaturing solution) the entire procedure of sample processing prior to LC-MS/MS (i.e., tryptic digestion and peptide clean-up/desalting) occurs in automated fashion on-line. The entire workflow occurs in 50 minutes, compared to the current state-of-art in ultrasensitive proteomics (i.e., FASP workflow) that is more than five times longer (approximately 6 hours) (Fig. S1), substantially reducing sample losses and significantly increasing throughput. Additionally, since the digestion process is automated, labor time is significantly reduced and each individual sample spends a fraction of the time at elevated temperatures reducing unwanted side reactions. A disadvantage of this approach is that digestion is now carried out serially, reducing the time savings for larger sample batches. Work is in progress to add a second digest column and SPE to the system, allowing for digestion of the subsequent sample during LC-MS/MS analysis of the preceding sample and thus fully exploiting the reduced sample handling time for increased throughput. This significant advance in throughput should be of particular benefit in enabling ultrasensitive proteomics studies with large cohorts such as in clinical applications.

### Performance evaluation of high-throughput LCM-proteomics platform for ultrasensitive analysis

**Figure 2:**
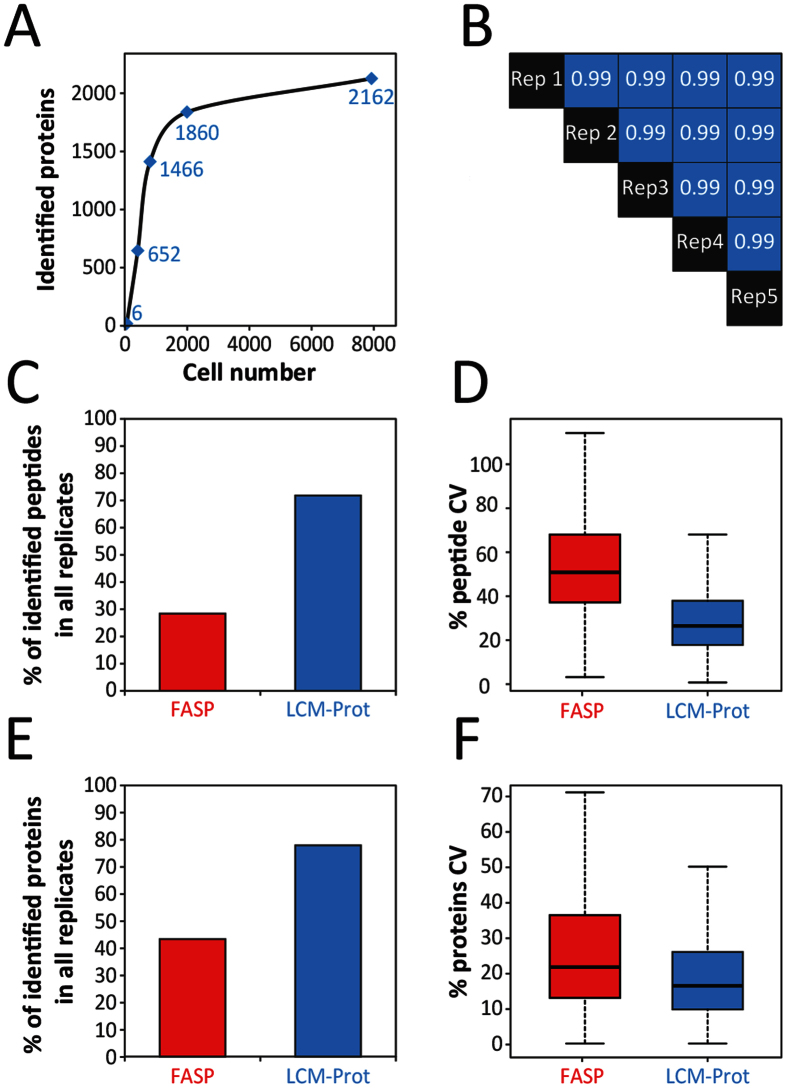
Performance evaluation of high-throughput LCM-proteomics platform on mass-limited LCM samples. (**A**) Chart showing number of identified proteins scales with number of cells represented in LCM sample. Increase in protein identifications begins to plateau after 2,000 cells. All the LCM cuts used for this figure were obtained from mice at the embryonic day 16.5. (**B**) Pearson’s correlation matrix demonstrating the reproducibility of the SNaPP platform at the protein level. Reproducibility was assessed utilizing 5 identical sample injections. The 5 replicate injections were performed from the same sample containing homogenate from 3 LCM cuts obtained at the post-natal day 7. The scatter plots representing the pairwise correlation plots for the proteins are shown in Fig. S2. (**C–F**) The comparison between FASP and our platform were performed in triplicate for each method and from LCM cuts obtained from mice at the post-natal day 28. (**C**) Percentage of identified peptides with measured intensity in all replicates for our platform and FASP. (**D**) Coefficient of variation for measured peptide abundance in our platform and FASP. (**E**) Percentage of identified proteins with measured intensity in all replicates for our platform and FASP. (**F**) Coefficient of variation for measured protein abundance in our platform and FASP. *LCM-Prot; LCM-proteomics platform*.

To evaluate the sensitivity of our platform for analyzing LCM samples, we injected samples of varying cell counts from 50 to 8,000 cells (Fig. 2A, Dataset S1). As expected, only few proteins were identified at 50 injected cells and increasing the number of injected cells increased the number of identified proteins. After 2,000 injected cells, the number of new protein identifications started to plateau, increasing by only 14% with a 4-fold higher amount of injected cells (i.e., 8,000 cells). Next, we evaluated the reproducibility of our platform by making five replicate injections of 2,000 cells each, originating from the same sample. Figure 2B, Fig. S2 and Dataset S1, show that injection replicates are highly correlated with Pearson correlations greater than 0.99, demonstrating the reproducibility of the platform for sub-microgram samples. Currently, the number of studies coupling microdissection and proteomics is limited; however, FASP has been shown to achieve similar protein coverage to our platform for samples containing as few as 3,000 cells^35^. Despite good protein coverage, FASP entails a complex multi-step workflow, long processing times (over 7 hrs of sample preparation time, including protein digest, for FASP relative to less than 1.5 hrs sample preparation time, for our platform) and concerns regarding its reproducibility have been suggested in literature^18^,^19^,^20^. Thus, we compared 3 equivalent samples processed with FASP and with our platform and evaluated the reproducibility of the resultant data both at the peptide and protein level. Figure 2C shows reproducibility of the peptide-level data across all three LC-MS analyses for each method. With FASP, less than 30% of identified peptides were observed in all 3 analyses (resulting in substantial missing data). Conversely, with our method more than 70% of peptides have a measured intensity in all replicates. Furthermore, measured intensities had lower variance when using our platform (Fig. 2D). Median coefficient of variance (CV) for peptides using FASP was 51%, compared to 27% with our platform. To ensure fair estimates of variance, CV’s were calculated using only peptides with intensities observed in all 3 datasets. A similar trend was observed at the protein level with less than 40% of proteins having a protein abundance value for all 3 FASP analyses, compared to nearly 80% of identified proteins with our platform (Fig. 2E) Protein abundances were determined using the LFQ (Label Free Quantification) approach as implemented in the Maxquant software package^9^. Using the LFQ approach to roll-up to protein abundances, as expected, the variance observed at the peptide-level was significantly reduced for both methods (Fig. 2F). However, the analysis with our platform still notably yielded improved quantitative reproducibility with a median CV of 17% across 1,219 proteins compared to 23% across only 647 proteins for FASP. This suggests more consistent sample handling is achieved by reducing manual operations and carrying out digestion and desalting online. The physico-chemical nature of the peptides resulting from FASP and our platform were found to be similar in terms of isoelectric point, molecular weight and hydrophobicity (Fig. S3). Nevertheless, some differences were observed between FASP and our platform generated peptides: the number of miscleaved peptides was found to be higher for our platform (Fig. S3); the number of semi-tryptic generated peptides was higher for the FASP method (data not shown); the number of contaminant attributed peptides was observed to be higher in FASP (Fig. S3). We speculate that the higher contamination (especially from human keratins) results from the multi-step manual handling required in FASP. Further, the number of miscleaved peptides can be controlled using this methodology by adjusting the flow rate or increasing the bed volume of the digestion column. Taken together, these results suggest that our platform enables improved in-depth, ultrasensitive global proteomics with high reproducibility for LCM samples. Therefore, we hypothesize that our platform will enable unique biological insights from LCM tissue samples not obtainable using other existing methods.

### Deep ultrasensitive proteomics of LCM alveolar tissue during lung development

**Figure 3:**
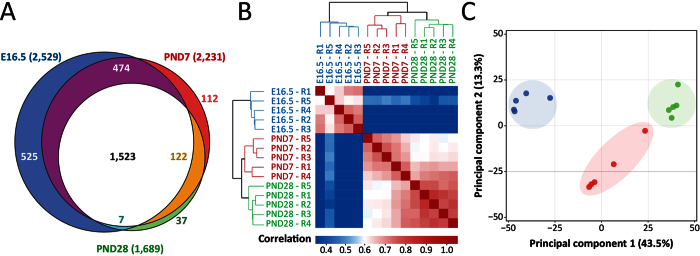
High-throughput LCM-proteomics platform enables effective comparative proteomics of mass-limited LCM samples. (**A**) Venn diagram of proteins quantified in at least three out of five replicate in one of the three developmental ages (E16.5, PND7, PND28) examined. (**B**) Pearson Correlation matrix and hierarchical clustering of the samples based on the protein LFQ intensities. (**C**) Principal components analysis (PCA) of the LCM samples at the three developmental ages (E16.5, PND7, PND28) examined; the percentage in the parenthesis represents the percentage of variance explained by the first and the second Principal Component (PC1 and PC2).

To demonstrate the utility of our approach for providing biological insights we applied it to analyze microdissected alveolar tissues to investigate the molecular mechanisms underlying normal lung alveolarization which remains poorly understood, but is critical for improving diagnosis and treatment outcomes during early lung growth and functional development. Lung alveolarization is a complex biological process involving various finely tuned and temporally and spatially resolved mechanisms^29^,^31^. Lung air-exchange tissue formation begins during the canalicular stage of development, from the embryonic day 16.5 (E16.5) to E17.5, in mice when each terminal airway duct starts to form an acinus^28^. During the saccular stage, from E17.5 to the post-natal day 5 (PND5), clusters of sacs are formed on the terminal bronchioles. As the interstitium between sacs is thinning, capillarization and cell differentiation occur. The cells composing the terminal sacs differentiate into Type I pneumocytes (Type I cells) involved in gas exchange and into Type II surfactant producing pneumocytes (Type II cells)^32^,^36^. Finally, during the last step of lung development: the alveolar stage (PND5-PND30), alveoli are formed from the terminal endings of the sacculi, their size increases and secondary septa are formed^28^,^37^. To date, the vast majority of global molecular profiling studies on normal lung development have been performed on whole lung lysates utilizing almost exclusively transcriptomics^31^,^38^,^39^; with only a single study in the literature^32^ reporting proteomics analysis of whole lung lysate during lung development^30^. To our knowledge, no global molecular profiling study (either proteomics or transcriptomics) has focused on the spatially-resolved analysis of the pathways/processes coordinating normal alveolar formation. In the present work, we have analyzed the ontogeny of protein changes in micro-dissected alveolar tissue (containing 4,000 cells) located at the termini of the respiratory tree during normal lung development. LCM samples from mouse lungs were obtained from three distinct developmental ages; the canalicular stage at E16.5, early alveolar stage at PND7 and late alveolar stage at PND28. The micro-dissected alveolar tissues contain numerous cell types, including Type I and Type II cells, endothelial cells, fibroblasts, lymphatic cells, myofibroblasts and (postnatally) increasing numbers of immune cells that interact during alveologenesis. For each time point, five equivalent micro‐dissected biological replicates originating from five different animals were analyzed using our platform. Across all the samples analyzed a total of 3,446 protein groups were identified with at least two peptides and a false discovery rate below 1% (Sup. Table S1). 2,800 of these were found with quantitative information in at least three out of the five replicates for at least one of the selected time points: 2,529 for E16.5; 2,231 for PND7; and 1,689 for PND28 (Fig. 3A; Sup. Table S1). The proteome coverage reported here represents one of the deepest for small-scale samples less than 5000 cells equivalent to or better than prior reports^12^,^16^,^35^. Pearson correlation analysis, shown in Fig. 3B, indicates that the samples within each time group were correlated with correlation values ranging from 0.60 to 1.00. As expected, the samples from the canalicular stage were less correlated to the samples from the alveolar stages (correlation values ranging from 0.26 to 0.50) than the samples from the two alveolar stages were to each other (correlation values ranging from 0.58 to 1.00). Similarly, the hierarchical clustering shows that samples originating from PND7 and PND28 were more closely related than the samples from E16.5. Nevertheless, the two alveolar samples were still distinguishable by the developmental age when collected. A principal component analysis performed on the generated dataset indicated that the first component alone (explaining 43.5% of the variance) was sufficient to differentiate the samples by developmental age (Fig. 3C). Taken together, these results show that our LCM-proteomics platform enables efficient reproducible detection of sample type-specific protein signatures from small sample concentrations (4,000 cells/sample in this case).

### Function-specific remodeling of alveolar proteome during lung development

**Figure 4:**
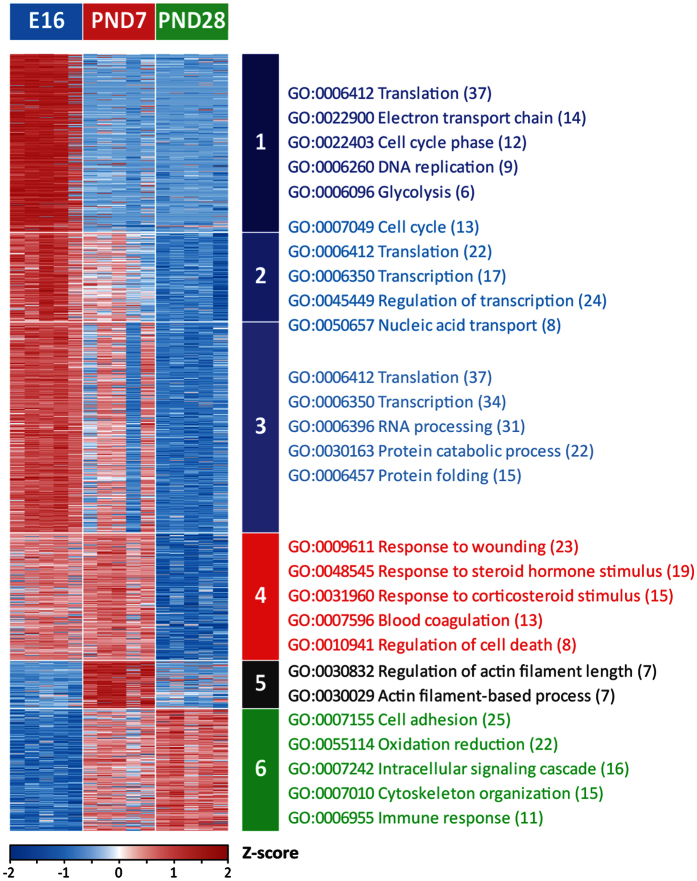
Proteins and biological functions significantly changing during alveolarization. The heatmap shows the 1,369 proteins that are changing in abundance over time (one-way ANOVA pvalue < 0.01). The color scale of the heatmap represents Z-scores of log_2_(normalized intensities). K-means clustering algorithm was used to classify the proteins into six clusters depending on their temporal behavior. For each cluster, the NIH DAVID Bioinformatics Resource was used to perform functional enrichment of the Biological Function GOs. Manually curated enriched Biological Functions are represented.

To elucidate the pathways/processes coordinating normal alveolar formation, we subjected the proteomics data to an ANOVA analysis. 1,369 of the quantifiable proteins (approximately 49%) were found to significantly change in abundance over time (one-way ANOVA pvalue < 0.01). For these proteins a k-mean (k = 6) clustering of normalized intensities revealed various protein abundance behaviors across the selected developmental ages (Fig. 4., Sup. Table S1). All the behaviors described below were further confirmed by pairwise Student tests (at least 70% of the proteins belonging to a given cluster had a t-test pvalue < 0.05 for the described behaviors, Sup. Table S1). The proteins of cluster 1 (314 proteins) were found to be more abundant at the canalicular stage than during either alveolar stage. Similarly, the proteins belonging to clusters 2 and 3 were found to be more abundant at the canalicular stage and decreased in abundance over time during the alveolar stage (530 proteins). The proteins of cluster 4 (225 proteins) were found lower in abundance in PND28 compared to the two earlier developmental ages (E16.5 and PND7). Cluster 5 (85 proteins) includes proteins that were more abundant at PND7 than at either E16.5 or PND28. Finally, the proteins of cluster 6 (215 proteins) were lower in abundance at the canalicular stage (E16.5) than at the alveolar stage (PND7 and PND28).

We performed a Gene Ontology (GO) enrichment analysis for each one of the above clusters using the DAVID bioinformatics resources^40^. Lists of manually curated Biological Process GOs enriched within each cluster are shown in Fig. 4. The biological processes that were enriched in the clusters 1 to 3 were mainly related to cell proliferation, energy production and nucleic acid and protein production suggesting that the cells contained in tissue harvested at E16.5 were more proliferative than post-natal cells. Cluster 4 was enriched in proteins related to signaling in response to wounding, hormonal stimuli or regulating cell death suggesting a regulatory reorganization between the developmental ages PND7 and PND28. Notably, various pro-inflammatory and pro-proliferative proteins were present in this cluster. For example, PRDX1 was recently shown to promote inflammation by increasing the abundance of pro-inflammatory cytokines^41^. Another example is the co-repressor carboxyl-terminal-binding protein (CtBP1) which is known for its proliferative role during tumorigenesis^42^ and to strongly interact with two of the key regulators of lung ontogeny: Foxp2 and Foxp1^43^,^44^. The biological functions enriched in cluster 5, which includes proteins higher in abundance at PND7, were all related to actin polymerization and de-polymerization; appearing to indicate cytoskeleton-driven tissue reorganization. Notably, the intermediate filament protein vimentin^45^,^46^ and the actin de-polymerization protein cofilin-1^47^,^48^ which are often used as Epithelial-Mesenchymal Transition (EMT) markers are higher at PND7 compared to E16.5 and PND28 (T-test pvalue < 0.01)^48^,^49^. The functions enriched in the proteins that were higher in abundance postnatally relative to in-utero (cluster 6) include oxidative stress response, likely upregulated as exposure to air with respiration has begun, and immune response related proteins, probably triggered by the exposure of the lungs to microorganisms in the ambient environment after birth or recruitment of bone marrow-derived cells into the lung. Other biological functions such as cell adhesion and cytoskeleton organization were also found to be enriched.

### A complex network of temporal regulatory control directs normal lung development

The complex succession of temporally defined events observed strongly supports the existence of a finely tuned regulatory network during development. Thus, we next extracted using the manually curated database available in Ingenuity Pathway Analysis the 396 transcription/translation regulators and signaling molecules that were quantifiable in our dataset (i.e., transcription factors [179 total proteins]; translation factors [48 total proteins]; kinases, phosphatases, growth factors, G-protein coupled receptors [169 total proteins]) and specifically examined their coordinated temporal expression during lung development. We note that these molecules are often challenging to detect and quantify because of their low abundance^50^ in samples, nevertheless our ultrasensitive platform enabled their analysis here. Utilizing a one-way ANOVA, 305 of the 396 regulatory and signaling proteins were significantly (pvalue < 0.05) changing in abundance over time, representing ~77% of regulatory and signaling molecules detected. More conservatively, at a pvalue < 0.01, 220 proteins (approximately 56% of regulatory and signaling molecules detected) were significantly changed and further at a pvalue < 0.001, 164 proteins (approximately 41% of regulatory and signaling molecules detected) were significantly changed. The large fraction of regulatory and signaling molecules changing reflects the significant architectural/regulatory and functional remodeling that must take place during lung development to ensure appropriate respiratory functions.

**Figure 5:**
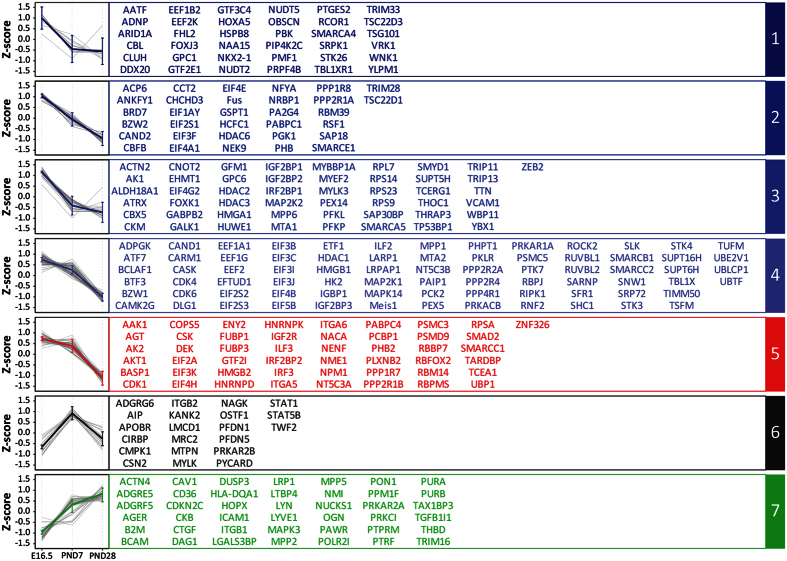
Regulatory and signaling proteins mediating alveolar formation. 305 proteins significantly changing over time (one-way ANOVA pvalue < 0.05) and annotated as a transcription/translation regulator or signaling molecule in the curated Ingenuity Pathway Analysis (IPA) database were clustered in 7 temporal behavioral groups using K-means clustering algorithm. On the left, the colored lines represent the average Z-scores of the cluster centroid over time. The error bars represent the Standard Error; the grey lines are the average of each individual protein belonging to a given cluster. Names of transcription/translation regulators and signaling molecules present in each cluster are written in the corresponding table.

Temporal expression analysis of significantly changing proteins defined seven groups of coordinated expression patterns supporting further the notion that a complex network of temporal regulatory control drives appropriate normal lung development (Fig. 5). Prior to this spatially-resolved proteomic profiling of the developing alveoli, only few of the low-abundance transcription factors were shown to be affected in abundance at the protein level. Notably, key regulatory proteins in lung organogenesis are described below.

**Figure 6:**
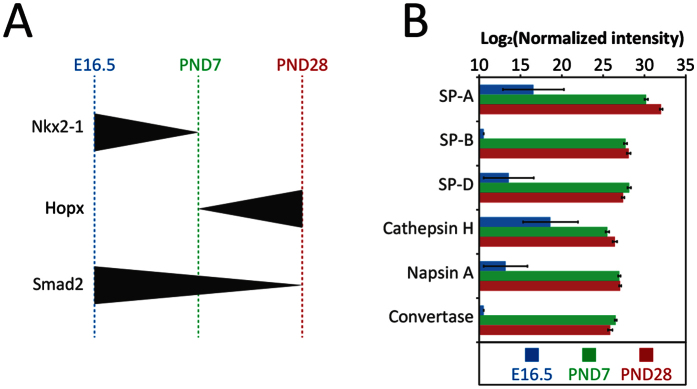
Known protein abundance patterns confirmed by LCM-proteomics platform during alveolarization. (**A**) Representation of abundance evolution for known transcription factors involved in lung development. Nkx2-1: highest expression at E16.5; Hopx: highest expression at PND28; Smad2: highest expression at E16.5. (**B**) Surfactant proteins and surfactant maturation associated proteins abundance at E16.5, PND7 and PND28; the errors bars represent the standard error.

The homeodomain-containing transcription factor Nkx2-1 (also known as TTF-1) is a key regulator of early lung morphogenesis, regulating a large network of genes important for lung development^51^. In line with this, we observed Nkx2-1 to be most abundant at the earliest time point of development (E16.5) in our analysis (T-test pvalue < 0.05, present in Cluster 1 in Figs 5 and 6A). AGER/RAGE is a receptor previously shown to be involved in idiopathic pulmonary fibrosis by triggering a TGF-β-dependent epithelial to myofibroblast transition localized in the alveolar region^52^. AGER/RAGE is known to be constitutively expressed in a wide range of organs during development with its expression in most organs down-regulated in adults, except in lungs where its selectively expressed in type I cells^52^,^53^ and its transcription remains high in adults^54^. Our alveolar region-specific protein data also showed AGER/RAGE abundance higher at PND28 which is the nearest condition to the adulthood compared to the earlier E16.5 and PND7 development time-points (T-test pvalue < 0.04; Cluster 7 in Fig. 5). Smad2 is also part of the TGF-β signaling pathway and was previously shown to involved in normal lung development as well as EMT transition inducing idiopathic pulmonary fibrosis^55^. During normal development, Smad2 mRNA abundance was previously described to significantly decrease over time in the lungs from E15 to PND28^56^. In our dataset a similar trend was observed: Smad2 was found higher in abundance at E16.5 relative to PND7 and PND28 and higher in PND7 compared to PND28 (T-test pvalue < 0.01; Cluster 5 in Figs 5 and 6A). Hopx is a homeodomain-containing protein that is involved in the type I type II cell differentiation from multipotent progenitors and becomes restricted to type I cells during development^57^. We observed Hopx to be less abundant at E16.5 relative to post-natal samples (T-test pvalue < 0.05; Cluster 7 in Figs 5 and 6A) in agreement with prior reports^31^. The receptor GPR116 (also known as Adgrf5) is known to be present in surfactant producing Type II cells^58^ and participates in the regulation of surfactant homeostasis^58^,^59^,^60^. GPR116 as well as all the quantifiable surfactant proteins in our dataset (SP-A, SP-B, SP-D) and associated surfactant protein maturation enzymes (including Cathepsin H, Napsin-A, convertase ES-2)^61^,^62^,^63^ were found to increase through development consistent with prior surfactant protein biochemical and transcriptome observations^64^,^65^ (Fig. 6B). The above examples, concordant with prior reports relevant to lung development validates our dataset and demonstrates that our platform is sensitive enough to allow the detection of fine changes in small micro-dissected samples, even for low abundance proteins such as transcriptional regulators.

### Epigenetic regulation fine-tunes pre-natal developmental processes

The role of epigenetic regulation in development is increasingly appreciated. The general mechanisms of epigenetic regulation that lead to chromatin remodeling and subsequently control gene expression include those involving covalent modifications (e.g. DNA methylation, histone post-translational modifications) and those that do not utilize covalent modifications (e.g. ATP-dependent chromatin remodeling complexes). Several proteins regulating chromatin structure and organization via both approaches were observed to decrease from E16.5 to PND7 and PND28 in our analysis (Fig. 5). These include members of the SWI/SNF protein family (SMARCA4, A5, E1, B1, C1, C2), histone deacetylases (HDAC1, 2, 3, 6) and high mobility group proteins (HMGA1, B1, B2). Recent work has shown an important role for HDACs in lung development^66^ and the importance of DNA methylation is also emerging^66^,^67^; however the role of other epigenetic regulatory mechanisms in lung development remains unclear^68^.

The HMG proteins are chromatin binding proteins that regulate transcription by modulating the chromatin structure at target genes influencing the binding of regulatory factors^69^. Similarly the SWI/SNF protein family, an ATP-dependent nucleosome remodeling complex, also regulates gene expression by modulating the nucleosome structure at target genes^70^. Here our data suggests these additional epigenetic regulatory mechanisms play an active role in lung development particularly at early (pre-natal) time-points in alveolar development where our data also suggests cellular proliferation is high (Fig. 4). Taken together, the data suggests a mechanism where epigenetic processes fine tune the high rate of cell proliferation earlier in lung development that subsides with increasing lung maturation. The current study suggests that a multi-omics analysis on LCM tissues, employing greater temporal resolution than reported here, may provide deeper insight into the finely tuned mechanisms controlling lung development. Indeed such an effort is underway as part of the LungMAP consortium (www.lungmap.net) and promises to yield further novel insights.

## Conclusion

We present a simple automated proteomic workflow that significantly reduces sample handling by employing online digestion and desalting that enables sensitive and robust quantitative proteomics analysis of less than 5,000 cells from LCM samples with high reproducibility and throughput. Our high-throughput analysis of micro-dissected alveolar parenchyma containing 4000 cells yielded a deep proteome coverage (more than 3,400 proteins) and revealed function-specific remodeling of the alveolar proteome during development, with proliferation-related biological processes induced during the saccular stage (E16.5) while immune response and structural biological processes were induced postnatally. Our analysis also suggested that epigenetic regulation is critical for lung development preferentially fine-tuning early processes in development. The demonstrated utility of our LCM-proteomics approach for gaining biological insight should now broadly enable deep spatially-resolved proteomics from mass-limited LCM samples for applications requiring high reproducibility and throughput such as clinical studies.

## Methods

### Mice

All the animal procedures were approved by the Institutional Animal Care and Use Committee (IACUC) at the University of Alabama at Birmingham (UAB), and carried out in accordance with UAB Institutional Animal Care and Use Committee guidelines and regulations. The lung tissues of E16.5 mice used in this study were obtained from timed-pregnant C57BL/6 mice at Cincinnati Children’s Hospital Medical Center, and lungs were frozen in OCT immediately after removing from the fetal mouse. Lung tissues of postnatal mice used in this study were obtained from pups born to C57BL/6 timed-pregnant females purchased from Jackson Laboratories (Bar Harbor, ME). Pups were euthanized with isoflurane inhalation at the specified age (PND7, PND28). The lungs were inflated to capacity through the trachea cannula with a 50/50 v/v solution of Tissue-Tek OCT in RNAse-free PBS. Working in the tissue culture hood using sterile scissors, the heart-lung complex was excised aseptically en bloc from the chest cavity and placed on ice. The lungs were placed into TissueTek cryomolds with excess OCT and snap frozen in liquid nitrogen. Frozen OCT lung tissues were stored at −80 °C no longer then one week prior to LCM. The MICROM-HM550 cryostat (Thermo scientific) was cleaned with 100% ethanol, and tools and surfaces in contact with the tissue were cleaned with RNase Away (Molecular BioProducts; San Diego, CA) to prepare for cryosectioning. 16 μm sections were collected by cryostat sectioning at −20 °C onto PEN membrane glass slides (Arcturus PEN#LCM0522). Two tissue sections were collected on the same slide and 10–20 slides were cut per animal and stored at −80 °C. Forty μl of prechilled RNAlater-ICE (Ambion/Applied Biosystems) was pipetted directly onto the slide, and the slide was stored flat in a prechilled slide box at −80 °C.

### LCM sections

Using the ArcturusXT Microdissection system the cells were captured by cutting the region of interest into the CapSure^®^ LCM Caps then placed in 500 μl RNase-free microfuge tube and immediately frozen on dry ice or stored at −80 °C. All CapSure^®^ LCM Caps have a patented transfer film bonded to the lower cap surface. Using the ArcturusXT Systems an infrared laser pulses through the top of the cap during LCM and interacts with the transfer film, which then melts and bonds to the cells or regions of interest. The film absorbs the laser radiation – instead of the tissue or cell sample – creating a gentle, non-damaging microdissection that preserves the integrity of the captured material. Slides were processed one at a time, taking care that each slide was at room temperature for less than 20 min to protect RNA integrity. For this study, only lung alveoli were captured while avoiding airways and vessels. For each alveolar region per animal (*n* = 5–6), sampling was very robust (3–4 tissue faces per section, 2 sections per slide, 10 slides) and areas ranging from 3.5 to 4 million μm^2^ were collected. All alveoli were collected for each section on each slide until 4 million μm^2^ were collected and sampling ceased. For each animal the entire alveoli sample was collected in a single cap when all LCM sampling occurred in a single session. If a break occurred during capture, remaining sample was collected into a new cap and the contents were later pooled for further analysis. When sample collection was complete, the tube was frozen at −80 °C and stored or immediately processed for RNA isolation using the RNeasy Plus Micro Kit (Qiagen) using gDNA eliminator columns.

### Protein extraction from the LCM cap

Samples were prepared by adding the following extraction buffer: 20 μL of 8 M urea, 5 mM DTT in 50 mM ammonium bicarbonate directly to the LCM caps. 4% SDS was added in this buffer when FASP digestion was used. The buffer was aspirated repeatedly to lose the tissue piece and allow its transfer: to a fresh Total Recovery LC-Vial (Waters) for the samples analyzed with our platform; or directly in the filtration unit for FASP. The samples were then sonicated in a Hielscher UTR200 bath sonicator for 20 sec and incubated at 37 °C for 30 min to extract and denature proteins. Protein extracts for SNaPP were prepared in a single batch process and stored at −80 °C until being loaded into the 4 °C autosampler of the SNaPP system for processing.

### FASP sample preparation

Three equivalent biological tissues were prepared by the FASP method using commercial kit (FASP Protein digestion Kit, Expedeon) following the supplier recommendations. Briefly, after the denaturation step previously described the denaturation buffer was removed by centrifugation at 14,000 g for 15 min. The sample was alkylated using 100 μL of 50 mM iodoacetamide in urea for 30 min in the dark, and excess alkylation reagents were eliminated by washing twice with 100 μL of 8 M urea solution (at 14,000 g for 15 min) and two more times with 50 mM ammonium bicarbonate (at 14,000 g for 15 minutes). The spin filters were then transferred to clean tubes and 50 μL of digestion solution containing trypsin was added to the filters. The protein to enzyme ratio was estimated to be 50:1. The top of the tubes were wrapped in parafilm to minimize evaporation and the tubes were placed for 4 h at 37 °C under 500 rpm agitation in a Thermomixer (Eppendorf). The peptides were concentrated to 20 μL using a speedvac vacuum concentrator (Thermo Scientific) and stored at −80 °C prior LC-MS/MS analysis (Fig. S1).

### LCM-proteomics platform

After extraction, samples were diluted to a final volume of 60 μL using 50 mM ammonium bicarbonate prior injection. Our online platform is equipped with a Leap autosampler allowing multiple samples to be queued and stored at 4 °C prior to injection and allowing the system to run unattended. Digestion was accomplished by passing the solution through a 150 μm ID column packed with immobilized trypsin beads from Poroszyme (Thermo Scientific) 10 μM diameter at a flow rate of 500 nL/min. Eluent was then captured on a trap column. C18, 5 μm, porous (300 Å) packed to 5 cm in length in a 100 μm i.d. capillary. Following digestion, the IMER column is washed with 25 μL of 50:50 Acetonitrile/water to reduce sample carryover. Digestions were carried out as described previously^27^, with the following changes: the analytical gradient pump was replaced by a Dionex UltiMate 3000 RSLC nanopump (Thermo Scientific) capable of achieving 12 K psi backpressures and allowing the operation of 75 cm capillary columns with 50 μm i.d. (Fig. 1).

### Liquid chromatography-mass spectrometry (LC-MS)

To increase protein coverage the total gradient time was extended to 300 min using an in-house packed, 50 μm i.d. fused silica capillary columns (Polymicro Technologies), 75 cm in length. Columns were slurry packed with 3 μm, porous (300 Å), Jupiter C_18_ packing material (Phenomenex) using a 1 cm sol-gel frit for retaining media^71^. Buffer A was water with 0.1% formic acid, and mobile phase B was acetonitrile with 0.1% formic acid. The separation gradient started at 5% mobile phase B increasing to 8% B at 6 minutes, 12% at 60 minutes, 35% at 225 minutes, 60% at 291 minutes, and 75% at 300 minutes. Carryover on the analytical column is addressed by running a “washing” gradient which ramps to 35% B twice, followed by a ramp to 95% B and then 2 more ramps to 35% B over 25 minutes. The system was coupled to a QExactive Plus mass spectrometer (Thermo Scientific) using a custom ESI interface comprised of 3 cm, chemically etched emitters coupled to the LC column using stainless steel unions (VICI Valco)^72^. Mass spectra were collected from 400–2,000 m/z at a resolution of 70 k followed by data dependent HCD MS/MS at a resolution of 17.5 K for the ten most abundant ions. For analysis of samples prepared by FASP, the entire 20 μL sample was injected onto the SPE bypassing the IMER column of the system and facilitating online SPE.

### Data analysis

Mass spectrometric raw data were analyzed in MaxQuant, version 1.5.2.8. with a false discovery rate set at 0.01^8^. Proteins were identified with at least 2 peptide of a minimum length of 6 amino acids by searching against the *Mus musculus* Uniprot database (UniprotKB, downloaded in 2015). Carbamidomethylation was set as fixed modification and N-terminal Acetylation and oxidation of methionine were included as dynamic modifications. Intensities were used for quantification at the peptide level for the comparison of our platform and FASP. LFQ quantification^9^ was used for protein quantification. For the comparison of our platform’s analysis of microdissected tissues at different ages, the individual intensities were log2 transformed and median normalized. The missing values were imputed by the minimum value of the resulting table divided by two. Two-tailed distribution homoscedastic T-Tests were performed in Microsoft Excel 2010. The Pearson’s correlation, K-mean clustering and hierarchical clustering were performed in R using the Stats package. The PCA was performed in R (version 3.2.2) using the ‘mixOmics’ package. GO enrichments were performed using DAVID bioinformatics resources^40^ only the groups with at least 5 proteins and a pvalue < 0.05 were considered enriched and are shown in the figures. The figures were generated in R or Microsoft Excel 2010 and visually adjusted in Adobe Illustrator (version 16.0.5).

## Additional Information

**Accession codes**: Mass spectrometric raw data were deposited in MassIVE (https://massive.ucsd.edu/) repository a member of the proteomeXchange consortium (http://www.proteomexchange.org/) under the accession MSV000079850. The identified peptide lists, the identified protein, and quantified proteins lists with the protein expression values and the associated statistics are attached to this manuscript in the supplementary Dataset S1.

**How to cite this article**: Clair, G. *et al*. Spatially-Resolved Proteomics: Rapid Quantitative Analysis of Laser Capture Microdissected Alveolar Tissue Samples. *Sci. Rep.* **6**, 39223; doi: 10.1038/srep39223 (2016).

**Publisher's note:** Springer Nature remains neutral with regard to jurisdictional claims in published maps and institutional affiliations.

## Supplementary Material

Supplementary Information

Supplementary Dataset 1

## References

[b1] Larance M. & Lamond A. I. Multidimensional proteomics for cell biology. Nat. Rev. Mol. Cell Biol. 16, 269–280 (2015). 25857810 10.1038/nrm3970

[b2] Datta S. . Laser capture microdissection: Big data from small samples. Histol. Histopathol. 30, 1255–69 (2015). 25892148 10.14670/HH-11-622 PMC4665617

[b3] Longuespée R. . Tissue proteomics for the next decade? Towards a molecular dimension in histology. OMICS 18, 539–52 (2014). 25105455 10.1089/omi.2014.0033

[b4] Heeren R. M. A., Smith D. F., Stauber J., Kükrer-Kaletas B. & MacAleese L. Imaging mass spectrometry: hype or hope? J. Am. Soc. Mass Spectrom. 20, 1006–14 (2009). 19318278 10.1016/j.jasms.2009.01.011

[b5] Lanekoff I., Stevens S. L., Stenzel-Poore M. P. & Laskin J. Matrix effects in biological mass spectrometry imaging: identification and compensation. Analyst 139, 3528–32 (2014). 24802717 10.1039/c4an00504j PMC4078919

[b6] Scheltema R. A. . The Q Exactive HF, a Benchtop mass spectrometer with a pre-filter, high-performance quadrupole and an ultra-high-field Orbitrap analyzer. Mol. Cell. Proteomics 13, 3698–708 (2014). 25360005 10.1074/mcp.M114.043489 PMC4256516

[b7] Eckhard U., Marino G., Butler G. S. & Overall C. M. Positional proteomics in the era of the human proteome project on the doorstep of precision medicine. Biochimie 122, 110–8 (2016). 26542287 10.1016/j.biochi.2015.10.018

[b8] Cox J. & Mann M. MaxQuant enables high peptide identification rates, individualized p.p.b.-range mass accuracies and proteome-wide protein quantification. Nat. Biotechnol. 26, 1367–72 (2008). 19029910 10.1038/nbt.1511

[b9] Cox J. . Accurate proteome-wide label-free quantification by delayed normalization and maximal peptide ratio extraction, termed MaxLFQ. Mol. Cell. Proteomics 13, 2513–26 (2014). 24942700 10.1074/mcp.M113.031591 PMC4159666

[b10] Altelaar A. F. M. & Heck A. J. R. Trends in ultrasensitive proteomics. Curr. Opin. Chem. Biol. 16, 206–13 (2012). 22226769 10.1016/j.cbpa.2011.12.011

[b11] Kulak N. A., Pichler G., Paron I., Nagaraj N. & Mann M. Minimal, encapsulated proteomic-sample processing applied to copy-number estimation in eukaryotic cells. Nat. Methods 11, 319–24 (2014). 24487582 10.1038/nmeth.2834

[b12] Waanders L. F. . Quantitative proteomic analysis of single pancreatic islets. Proc. Natl. Acad. Sci. USA. 106, 18902–7 (2009). 19846766 10.1073/pnas.0908351106 PMC2765458

[b13] Liu N. Q. . Quantitative proteomic analysis of microdissected breast cancer tissues: comparison of label-free and SILAC-based quantification with shotgun, directed, and targeted MS approaches. J. Proteome Res. 12, 4627–41 (2013). 23957277 10.1021/pr4005794

[b14] Bark S. J. & Hook V. Differential recovery of peptides from sample tubes and the reproducibility of quantitative proteomic data. J. Proteome Res. 6, 4511–6 (2007). 17850064 10.1021/pr070294o

[b15] Gutstein H. B., Morris J. S., Annangudi S. P. & Sweedler J. V. Microproteomics: analysis of protein diversity in small samples. Mass Spectrom. Rev. 27, 316–30 (2008). 18271009 10.1002/mas.20161 PMC2743962

[b16] Hughes C. S. . Ultrasensitive proteome analysis using paramagnetic bead technology. Mol. Syst. Biol. 10, 757 (2014). 25358341 10.15252/msb.20145625 PMC4299378

[b17] Wiśniewski J. R., Zougman A., Nagaraj N. & Mann M. Universal sample preparation method for proteome analysis. Nat. Methods 6, 359–62 (2009). 19377485 10.1038/nmeth.1322

[b18] Nel A. J. M., Garnett S., Blackburn J. M. & Soares N. C. Comparative reevaluation of FASP and enhanced FASP methods by LC-MS/MS. J. Proteome Res. 14, 1637–42 (2015). 25619111 10.1021/pr501266c

[b19] An B., Zhang M., Johnson R. W. & Qu J. Surfactant-aided precipitation/on-pellet-digestion (SOD) procedure provides robust and rapid sample preparation for reproducible, accurate and sensitive LC/MS quantification of therapeutic protein in plasma and tissues. Anal. Chem. 87, 4023–9 (2015). 25746131 10.1021/acs.analchem.5b00350

[b20] Liebler D. C. & Ham A.-J. L. Spin filter-based sample preparation for shotgun proteomics. Nat. Methods 6, 785; author reply 785–6 (2009). 19876013 10.1038/nmeth1109-785a

[b21] Girelli A. M. & Mattei E. Application of immobilized enzyme reactor in on-line high performance liquid chromatography: a review. J. Chromatogr. B. Analyt. Technol. Biomed. Life Sci. 819, 3–16 (2005). 10.1016/j.jchromb.2005.01.031 15797515

[b22] Yamaguchi H. & Miyazaki M. Enzyme-immobilized reactors for rapid and efficient sample preparation in MS-based proteomic studies. Proteomics 13, 457–66 (2013). 23255229 10.1002/pmic.201200272

[b23] Hustoft H. K. . Open tubular lab-on-column/mass spectrometry for targeted proteomics of nanogram sample amounts. PLoS One 9, e106881 (2014). 25222838 10.1371/journal.pone.0106881 PMC4164520

[b24] Sun L., Zhu G. & Dovichi N. J. Integrated capillary zone electrophoresis-electrospray ionization tandem mass spectrometry system with an immobilized trypsin microreactor for online digestion and analysis of picogram amounts of RAW 264.7 cell lysate. Anal. Chem. 85, 4187–94 (2013). 23510126 10.1021/ac400523x PMC3635492

[b25] Shen Y. . High-efficiency nanoscale liquid chromatography coupled on-line with mass spectrometry using nanoelectrospray ionization for proteomics. Anal. Chem. 74, 4235–49 (2002). 12199598 10.1021/ac0202280

[b26] Luo Q. . More sensitive and quantitative proteomic measurements using very low flow rate porous silica monolithic LC columns with electrospray ionization-mass spectrometry. J. Proteome Res. 5, 1091–7 (2006). 16674098 10.1021/pr050424y

[b27] Huang E. L. . SNaPP: Simplified Nano-Proteomics Platform for reproducible global proteomic analysis of nanogram protein quantities. Endocrinology en20151821, doi: 10.1210/en.2015-1821 (2016). PMC4769369 26745641

[b28] Warburton D. . Lung organogenesis. Curr. Top. Dev. Biol. 90, 73–158 (2010). 20691848 10.1016/S0070-2153(10)90003-3 PMC3340128

[b29] Chinoy M. R. Lung growth and development. Front. Biosci. 8, d392–415 (2003). 12456356 10.2741/974

[b30] Bhattacharya S. & Mariani T. J. Systems biology approaches to identify developmental bases for lung diseases. Pediatr. Res. 73, 514–22 (2013). 23314295 10.1038/pr.2013.7 PMC3615902

[b31] Xu Y. . Transcriptional programs controlling perinatal lung maturation. PLoS One 7, e37046 (2012). 22916088 10.1371/journal.pone.0037046 PMC3423373

[b32] Cox B. . Integrated proteomic and transcriptomic profiling of mouse lung development and Nmyc target genes. Mol. Syst. Biol. 3, 109 (2007). 17486137 10.1038/msb4100151 PMC2673710

[b33] Crosetto N., Bienko M. & van Oudenaarden A. Spatially resolved transcriptomics and beyond. Nat. Rev. Genet. 16, 57–66 (2014). 25446315 10.1038/nrg3832

[b34] Piehowski P. D. . Sources of technical variability in quantitative LC-MS proteomics: human brain tissue sample analysis. J. Proteome Res. 12, 2128–37 (2013). 23495885 10.1021/pr301146m PMC3695475

[b35] Wiśniewski J. R., Ostasiewicz P. & Mann M. High recovery FASP applied to the proteomic analysis of microdissected formalin fixed paraffin embedded cancer tissues retrieves known colon cancer markers. J. Proteome Res. 10, 3040–9 (2011). 21526778 10.1021/pr200019m

[b36] Whitsett J. A. The molecular era of surfactant biology. Neonatology 105, 337–43 (2014). 24931326 10.1159/000360649 PMC4108987

[b37] Mund S. I., Stampanoni M. & Schittny J. C. Developmental alveolarization of the mouse lung. Dev. Dyn. 237, 2108–16 (2008). 18651668 10.1002/dvdy.21633

[b38] Mariani T. J., Reed J. J. & Shapiro S. D. Expression profiling of the developing mouse lung: insights into the establishment of the extracellular matrix. Am. J. Respir. Cell Mol. Biol. 26, 541–8 (2002). 11970905 10.1165/ajrcmb.26.5.2001-00080c

[b39] Kho A. T. . Age, Sexual Dimorphism and Disease Associations in the Developing Human Fetal Lung Transcriptome. Am. J. Respir. Cell Mol. Biol. doi: 10.1165/rcmb.2015-0326OC (2015) PMC4942221 26584061

[b40] Huang D. W., Sherman B. T. & Lempicki R. A. Systematic and integrative analysis of large gene lists using DAVID bioinformatics resources. Nat. Protoc. 4, 44–57 (2009). 19131956 10.1038/nprot.2008.211

[b41] Liu D. . Proteomic analysis of lung tissue in a rat acute lung injury model: identification of PRDX1 as a promoter of inflammation. Mediators Inflamm. 2014, 469358 (2014). 25024510 10.1155/2014/469358 PMC4082880

[b42] Barabutis N., Siejka A. & Schally A. V. Effects of growth hormone-releasing hormone and its agonistic and antagonistic analogs in cancer and non-cancerous cell lines. Int. J. Oncol. 36, 1285–9 (2010). 20372804 10.3892/ijo_00000613

[b43] Shu W. . Foxp2 and Foxp1 cooperatively regulate lung and esophagus development. Development 134, 1991–2000 (2007). 17428829 10.1242/dev.02846

[b44] Li S., Weidenfeld J. & Morrisey E. E. Transcriptional and DNA binding activity of the Foxp1/2/4 family is modulated by heterotypic and homotypic protein interactions. Mol. Cell. Biol. 24, 809–22 (2004). 14701752 10.1128/MCB.24.2.809-822.2004 PMC343786

[b45] Rock J. R. . Multiple stromal populations contribute to pulmonary fibrosis without evidence for epithelial to mesenchymal transition. Proc. Natl. Acad. Sci. USA. 108, E1475–83 (2011). 22123957 10.1073/pnas.1117988108 PMC3248478

[b46] Beyea J. A. . Growth hormone (GH) receptor knockout mice reveal actions of GH in lung development. Proteomics 6, 341–8 (2006). 16287172 10.1002/pmic.200500168

[b47] Park K.-S. & Gumbiner B. M. Cadherin-6B stimulates an epithelial mesenchymal transition and the delamination of cells from the neural ectoderm via LIMK/cofilin mediated non-canonical BMP receptor signaling. Dev. Biol. 366, 232–43 (2012). 22537493 10.1016/j.ydbio.2012.04.005 PMC3358420

[b48] Lamouille S., Xu J. & Derynck R. Molecular mechanisms of epithelial-mesenchymal transition. Nat. Rev. Mol. Cell Biol. 15, 178–96 (2014). 24556840 10.1038/nrm3758 PMC4240281

[b49] Bartis D., Mise N., Mahida R. Y., Eickelberg O. & Thickett D. R. Epithelial-mesenchymal transition in lung development and disease: does it exist and is it important? Thorax 69, 760–5 (2014). 24334519 10.1136/thoraxjnl-2013-204608

[b50] Smaczniak C. . Proteomics-based identification of low-abundance signaling and regulatory protein complexes in native plant tissues. Nat. Protoc. 7, 2144–2158 (2012). 23196971 10.1038/nprot.2012.129

[b51] Maeda Y., Davé V. & Whitsett J. A. Transcriptional control of lung morphogenesis. Physiol. Rev. 87, 219–44 (2007). 17237346 10.1152/physrev.00028.2006

[b52] Song J. S. . Inhibitory effect of receptor for advanced glycation end products (RAGE) on the TGF-β-induced alveolar epithelial to mesenchymal transition. Exp. Mol. Med. 43, 517–24 (2011). 21743278 10.3858/emm.2011.43.9.059 PMC3203242

[b53] Treutlein B. . Reconstructing lineage hierarchies of the distal lung epithelium using single-cell RNA-seq. Nature 509, 371–375 (2014). 24739965 10.1038/nature13173 PMC4145853

[b54] Brett J. . Survey of the distribution of a newly characterized receptor for advanced glycation end products in tissues. Am. J. Pathol. 143, 1699–712 (1993). 8256857 PMC1887265

[b55] Watanabe-Takano H. . DA-Raf-dependent inhibition of the Ras-ERK signaling pathway in type 2 alveolar epithelial cells controls alveolar formation. Proc. Natl. Acad. Sci. USA. 111, E2291–300 (2014). 24843139 10.1073/pnas.1321574111 PMC4050578

[b56] Alejandre-Alcázar M. A. . TGF-beta signaling is dynamically regulated during the alveolarization of rodent and human lungs. Dev. Dyn. 237, 259–69 (2008). 18095342 10.1002/dvdy.21403

[b57] Jain R. . Plasticity of Hopx(+) type I alveolar cells to regenerate type II cells in the lung. Nat. Commun. 6, 6727 (2015). 25865356 10.1038/ncomms7727 PMC4396689

[b58] Bridges J. P. . Orphan G protein-coupled receptor GPR116 regulates pulmonary surfactant pool size. Am. J. Respir. Cell Mol. Biol. 49, 348–57 (2013). 23590306 10.1165/rcmb.2012-0439OC PMC3824053

[b59] Fukuzawa T. . Lung surfactant levels are regulated by Ig-Hepta/GPR116 by monitoring surfactant protein D. PLoS One 8, e69451 (2013). 23922714 10.1371/journal.pone.0069451 PMC3726689

[b60] Niaudet C. . Gpr116 Receptor Regulates Distinctive Functions in Pneumocytes and Vascular Endothelium. PLoS One 10, e0137949 (2015). 26394398 10.1371/journal.pone.0137949 PMC4579087

[b61] Bühling F. . Gene targeting of the cysteine peptidase cathepsin H impairs lung surfactant in mice. PLoS One 6, e26247 (2011). 22022579 10.1371/journal.pone.0026247 PMC3192174

[b62] Woischnik M. . Cathepsin H and napsin A are active in the alveoli and increased in alveolar proteinosis. Eur. Respir. J. 31, 1197–204 (2008). 18216060 10.1183/09031936.00081207

[b63] Ruppert C. . Liver carboxylesterase cleaves surfactant protein (SP-) B and promotes surfactant subtype conversion. Biochem. Biophys. Res. Commun. 348, 1449–54 (2006). 16919595 10.1016/j.bbrc.2006.08.013

[b64] Khoor A., Stahlman M. T., Gray M. E. & Whitsett J. A. Temporal-spatial distribution of SP-B and SP-C proteins and mRNAs in developing respiratory epithelium of human lung. J. Histochem. Cytochem. 42, 1187–99 (1994). 8064126 10.1177/42.9.8064126

[b65] Kishore U. . Surfactant proteins SP-A and SP-D: structure, function and receptors. Mol. Immunol. 43, 1293–315 (2006). 16213021 10.1016/j.molimm.2005.08.004

[b66] Dakhlallah D. . Epigenetic regulation of miR-17~92 contributes to the pathogenesis of pulmonary fibrosis. Am. J. Respir. Crit. Care Med. 187, 397–405 (2013). 23306545 10.1164/rccm.201205-0888OC PMC3603596

[b67] Benlhabib H. & Mendelson C. R. Epigenetic regulation of surfactant protein A gene (SP-A) expression in fetal lung reveals a critical role for Suv39h methyltransferases during development and hypoxia. Mol. Cell. Biol. 31, 1949–58 (2011). 21402781 10.1128/MCB.01063-10 PMC3133366

[b68] Herriges M. & Morrisey E. E. Lung development: orchestrating the generation and regeneration of a complex organ. Development 141, 502–13 (2014). 24449833 10.1242/dev.098186 PMC3899811

[b69] Catez F. . Network of dynamic interactions between histone H1 and high-mobility-group proteins in chromatin. Mol. Cell. Biol. 24, 4321–8 (2004). 15121851 10.1128/MCB.24.10.4321-4328.2004 PMC400478

[b70] Furlan-Margaril M. & Recillas-Targa F. In Topics in Animal and Plant Development: From Cell Differentiation to Morphogenesis (ed. Chimal-Monroy J.) 221–247 at https://issuu.com/researchsignpost/docs/chimal_monroy_e-_book (2011).

[b71] Maiolica A., Borsotti D. & Rappsilber J. Self-made frits for nanoscale columns in proteomics. Proteomics 5, 3847–3850 (2005). 16130174 10.1002/pmic.200402010

[b72] Kelly R. T. . Chemically Etched Open Tubular and Monolithic Emitters for Nanoelectrospray Ionization Mass Spectrometry. Anal. Chem. 78, 7796–7801 (2006). 17105173 10.1021/ac061133r PMC1769309

