# Supplementary material for: Spatially-Resolved Proteomics: Rapid Quantitative Analysis of Laser Capture Microdissected Alveolar Tissue Samples
Source: Sci Rep. 2016 Dec 22;6:39223. doi: 10.1038/srep39223 (PMC5177886; doi:10.1038/srep39223)
Supplement: Supplementary Information [file srep39223-s1.pdf]

Spatially-Resolved Proteomics: Rapid Quantitative Analysis of Laser Capture Microdissected Alveolar Tissue Samples

Geremy Clair[1*], Paul Piehowski[1*], Teodora Nicola[2], Joseph Kitzmiller[3], Eric Huang[1], Erika Zink[1], Ryan Sontag[1], Danny Orton[1], Ron Moore[1], James Carson[4], Richard D. Smith[1], Jeffrey A. Whitsett[3], Richard A. Corley[1], Namasivayam Ambalavanan[2] and Charles Ansong[1#].

[*] Contributed equally

[#] Corresponding author

[1]Biological Science Division, Pacific Northwest National Laboratory, Richland, WA 99352

[2]Department of Pediatrics, University of Alabama at Birmingham, Birmingham, AL 35249

[3]Division of Pulmonary Biology, Cincinnati Children's Hospital Medical Center, Cincinnati, OH 45229

[4]Texas Advanced Computing Center, University of Texas at Austin, Austin, TX 78712

Supplemental Figure legends.

Figure S1. Comparison of time required for FASP strategy and current LCM-proteomics strategy. The sample preparation of our LCM-proteomics approach is more than five times shorter compared with the FASP protocol. *LCM-Prot; LCM-proteomics platform*.

Figure S2 - Pearson's correlation matrix demonstrating the reproducibility of the SNaPP platform at the protein level. Reproducibility was assessed utilizing 5 identical sample injections. The 5 replicate injections were performed from the same sample containing homogenate from 3 LCM cuts obtained at PND7. The blue shaded portion of the correlation matrix represent the Pearson correlation values for the protein LFQ intensities originating from each of the 5 replicate samples, the unshaded portion represent the corresponding pairwise correlation plot for the proteins.

Figure S3. Comparison of the biochemical properties of FASP and our LCM-proteomics platform generated peptides. Charts representing the frequency of peptides biochemical properties show that our platform generated peptides and FASP generated peptides are comparable in terms of (A) Isoelectric point , (B) Molecular weight and (C) Hydrophobicity (Gravy score). (D) The Venn diagram represent all the peptides detected only with FASP (in red), with both platforms (in purple), or only with our platform (in blue). All the peptides were considered (including the miscleavage-containing peptides and the semi-tryptic peptides). (E)The number of miscleavages is higher in our platform compared to FASP. (F) FASP generated

samples contain more keratin contaminant than our platform generated samples. *LCM-Prot; LCM-proteomics platform*.

Supplemental Dataset description.

Dataset S1. Peptide table, protein table and protein expression tables used for this study.

1.Proteins 50 cells	List of identified proteins for an equivalent of 50 cells analyzed - The sample originate from LCM cuts obtained at E16.5
2.Proteins 500 cells	List of identified proteins for an equivalent of 500 cells analyzed - The sample originate from LCM cuts obtained at E16.5
3.Proteins 1000 cells	List of identified proteins for an equivalent of 1000 cells analyzed - The sample originate from LCM cuts obtained at E16.5
4.Proteins 2000 cells	List of identified proteins for an equivalent of 2000 cells analyzed - The sample originate from LCM cuts obtained at E16.5
5.Proteins 8000 cells	List of identified proteins for an equivalent of 8000 cells analyzed - The sample originate from LCM cuts obtained at E16.5
6.Peptides repetition	List of identified peptides from the 5 repetition experiment - The sample is a pool from 3 cuts of ~4000 cells obtaine at PND7 - The expression values are MaxQuant peptide intensities
7.Proteins repetition	List of identified proteins from the 5 repetition experiment - The sample is a pool from 3 cuts of ~4000 cells obtaine at PND7 - The expression values are MaxQuant LFQ intensities
8.Peptides FASP vs LCM-Prot	List of identified peptides from the comparison between FASP and our LCM-Prot method - The sample originate from LCM cuts obtained at PND28 - The expression values are MaxQuant peptide intensities
9.Proteins FASP vs LCM-Prot	List of identified peptides from the comparison between FASP and our LCM-Prot method - The sample originate from LCM cuts obtained at PND28 - The expression values are MaxQuant peptide intensities
10.Identified peptides temporal	Identified peptides - The sample originate from LCM cuts obtained at E16.5, PND7 and PND28 - The expression values are MaxQuant Peptide intensities

11.Identified proteins temporal	Identified proteins with at least 2 unique peptides per protein identification - The sample originate from LCM cuts obtained at E16.5, PND7 and PND28 - The expression values are the RAW MaxQuant LFQ intensities
12.Quantified proteins temporal	Identified proteins present in at least 3 out of 5 replicates in any timepoint(quantifiable) - The sample originate from LCM cuts obtained at E16.5, PND7 and PND28 - The expression values shown are the MaxQuant LFQ intensities log2 transformed, median normalized and imputed.
13.Regulators and SM temporal	Subset of quantified transcriptional/translational regulators and signaling molecules (SM) - The sample originate from LCM cuts obtained at E16.5, PND7 and PND28 - The expression values shown are the MaxQuant LFQ intensities log2 transformed, median normalized and imputed.

Figure S1

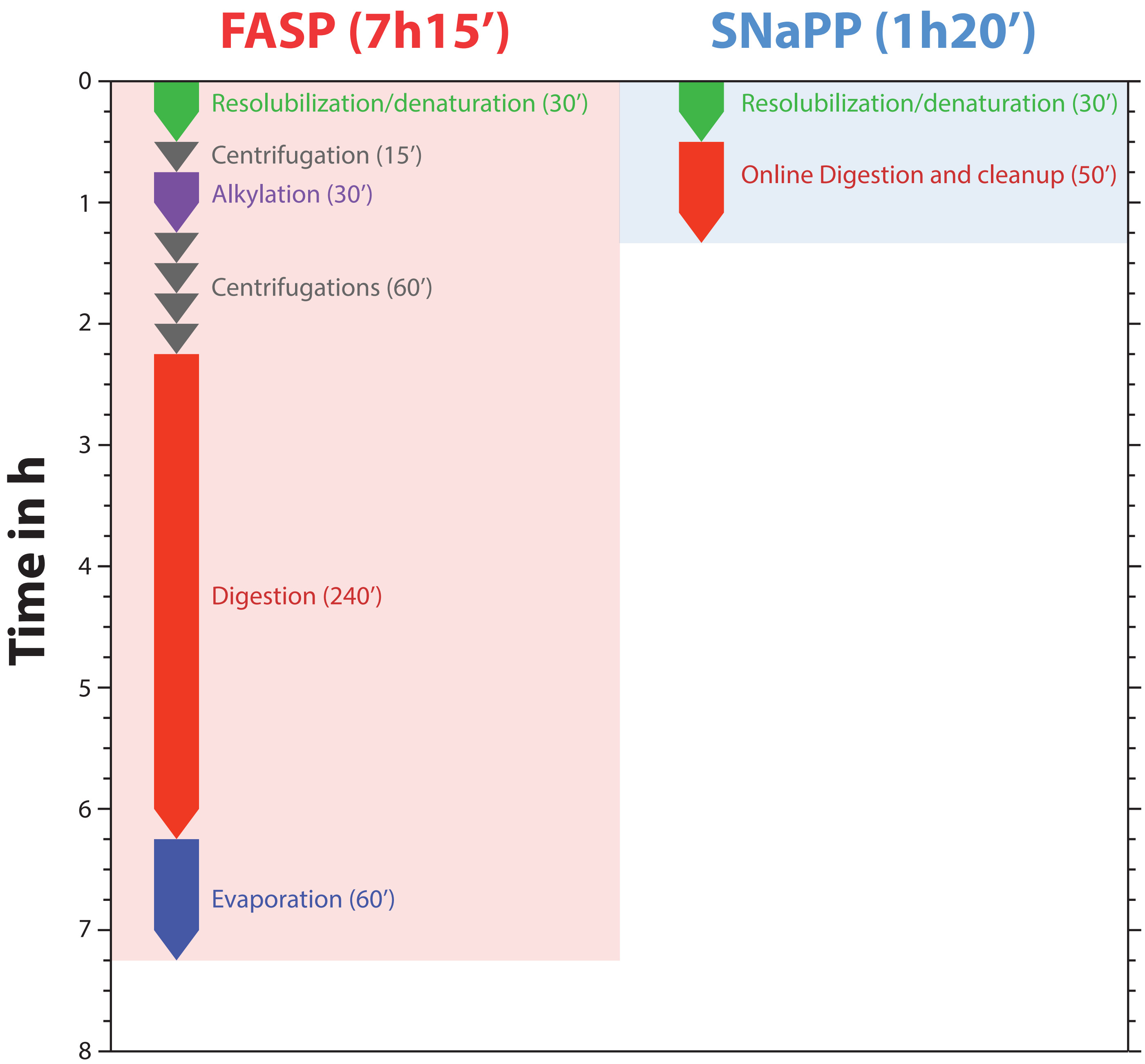
FASP (7h15’)
SNaPP (1h20’)
Time in h
0
1
2
3
4
5
6
7
8
Resolubilization/denaturation (30’)
Centrifugation (15’)
Alkylation (30’)
Centrifugations (60’)
Digestion (240’)
Evaporation (60’)
Resolubilization/denaturation (30’)
Online Digestion and cleanup (50’)

Figure S2

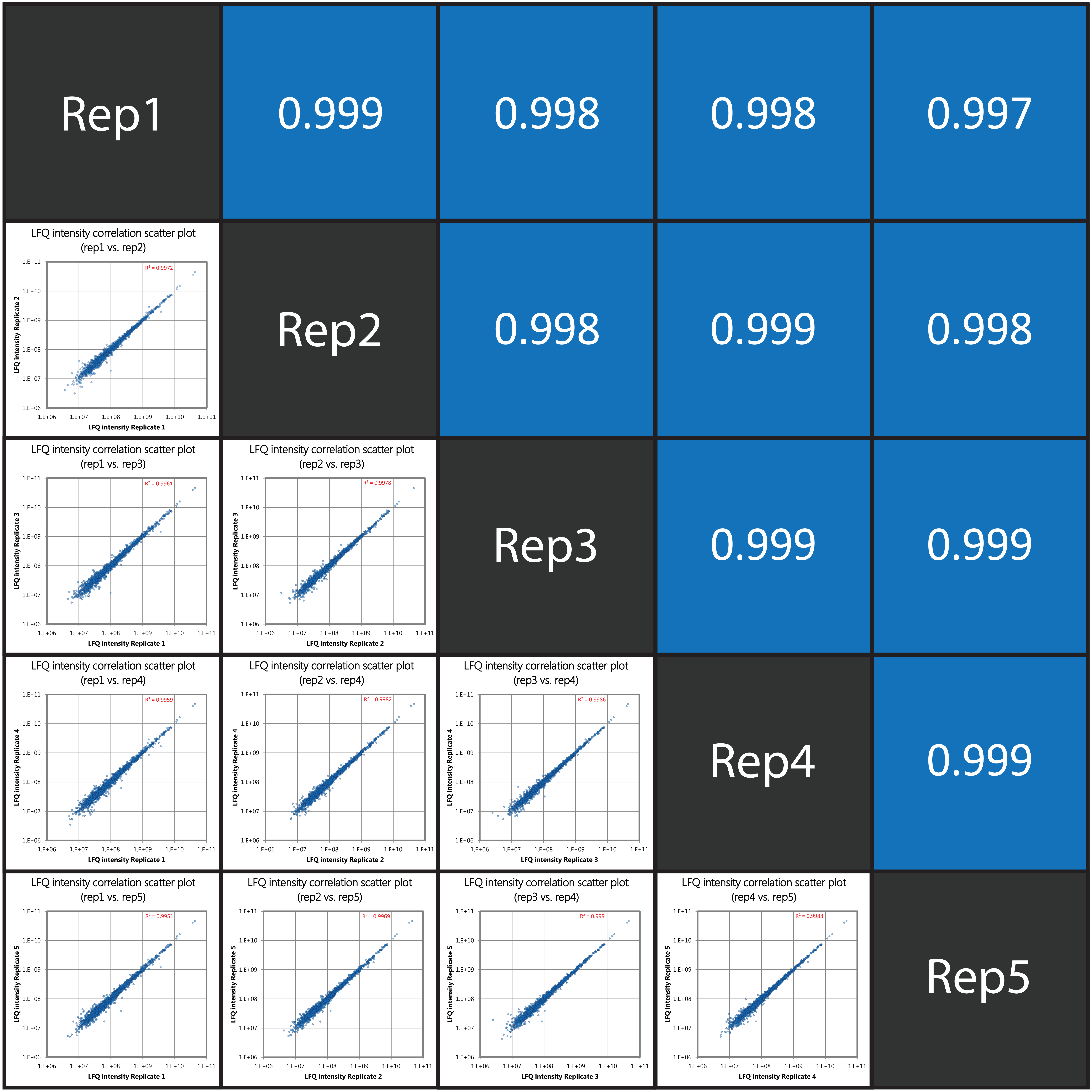
Rep1
0.999
0.998
0.998
0.997
LFQ intensity correlation scatter plot (rep1 vs. rep2)
R² = 0.9972
LFQ intensity Replicate 2
LFQ intensity Replicate 1
Rep2
0.998
0.999
0.998
LFQ intensity correlation scatter plot (rep1 vs. rep3)
R² = 0.9961
LFQ intensity Replicate 3
LFQ intensity Replicate 1
LFQ intensity correlation scatter plot (rep2 vs. rep3)
R² = 0.9978
LFQ intensity Replicate 3
LFQ intensity Replicate 2
Rep3
0.999
0.999
LFQ intensity correlation scatter plot (rep1 vs. rep4)
R² = 0.9959
LFQ intensity Replicate 4
LFQ intensity Replicate 1
LFQ intensity correlation scatter plot (rep2 vs. rep4)
R² = 0.9982
LFQ intensity Replicate 4
LFQ intensity Replicate 2
LFQ intensity correlation scatter plot (rep3 vs. rep4)
R² = 0.9986
LFQ intensity Replicate 4
LFQ intensity Replicate 3
Rep4
0.999
LFQ intensity correlation scatter plot (rep1 vs. rep5)
R² = 0.9951
LFQ intensity Replicate 5
LFQ intensity Replicate 1
LFQ intensity correlation scatter plot (rep2 vs. rep5)
R² = 0.9969
LFQ intensity Replicate 5
LFQ intensity Replicate 2
LFQ intensity correlation scatter plot (rep3 vs. rep4)
R² = 0.999
LFQ intensity Replicate 5
LFQ intensity Replicate 3
LFQ intensity correlation scatter plot (rep4 vs. rep5)
R² = 0.9988
LFQ intensity Replicate 5
LFQ intensity Replicate 4
Rep5

Figure S3

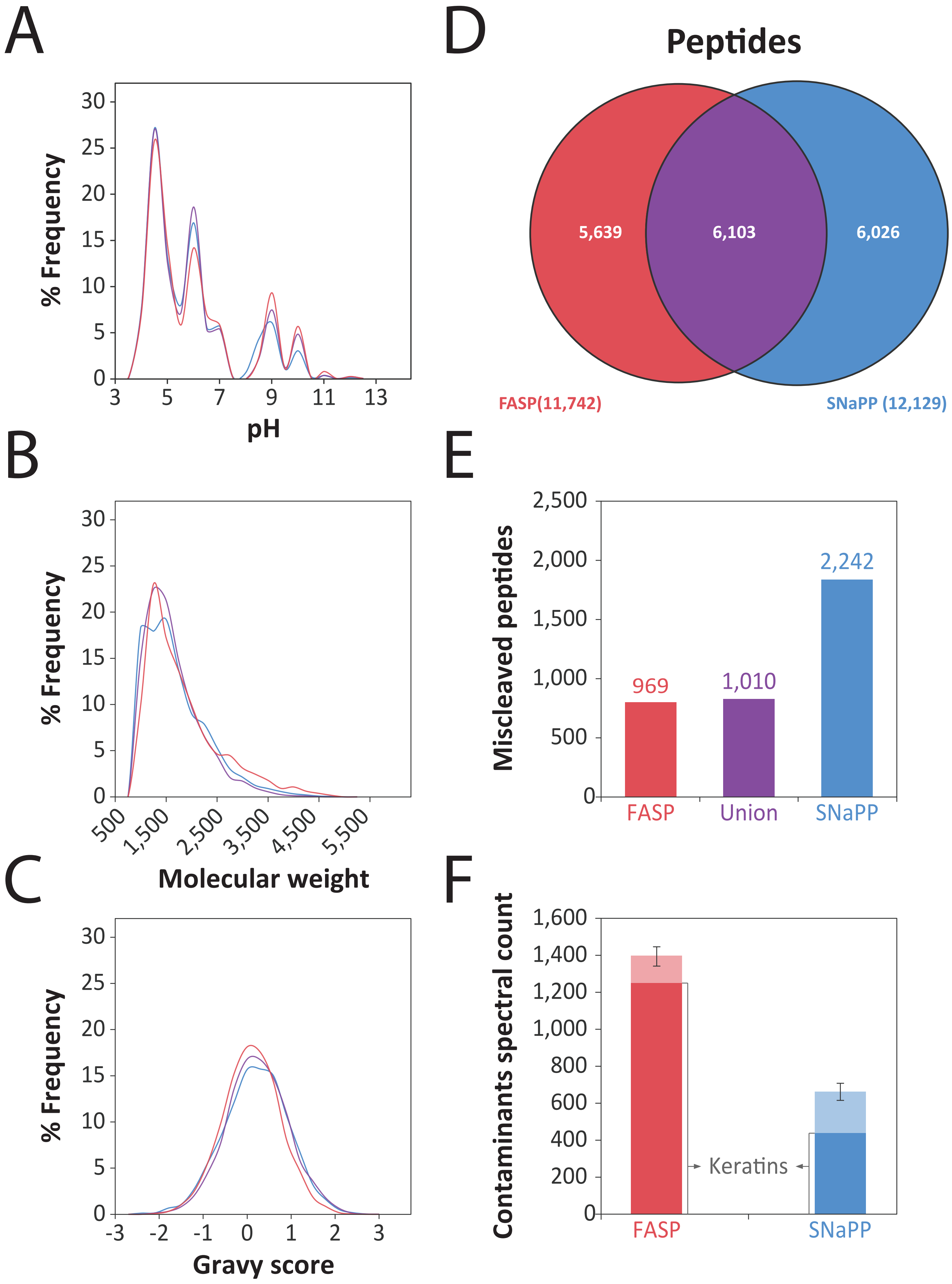

A
30
25
20
15
10
5
0
% Frequency
3
5
7
9
11
13
pH
B
30
25
20
15
10
5
0
% Frequency
500
1,500
2,500
3,500
4,500
5,500
Molecular weight
C
30
25
20
15
10
5
0
% Frequency
-3
-2
-1
0
1
2
3
Gravy score
D
Peptides
5,639
6,103
6,026
FASP(11,742)
SNaPP (12,129)
E
2,500
2,000
1,500
1,000
500
0
Miscleaved peptides
969
1,010
2,242
FASP
Union
SNaPP
F
1,600
1,400
1,200
1,000
800
600
400
200
0
Contaminants spectral count
Keratins
FASP
SNaPP
